# Cohort-level analysis of human *de novo* mutations points to drivers of clonal expansion in spermatogonia

**DOI:** 10.1101/2025.01.03.25319979

**Published:** 2025-06-20

**Authors:** Vladimir Seplyarskiy, Mikhail A Moldovan, Evan Koch, Prathitha Kar, Matthew DC Neville, Raheleh Rahbari, Shamil Sunyaev

**Affiliations:** 1 Department of Biomedical Informatics, Harvard Medical School, Boston, MA, USA; 2 Brigham and Women’s Hospital, Division of Genetics, Harvard Medical School, Boston, MA, USA; 3 Cancer, Ageing and Somatic Mutation, Wellcome Sanger Institute, Hinxton, United Kingdom

## Abstract

In renewing tissues, mutations conferring selective advantage may result in clonal expansions^1–4^. In contrast to somatic tissues, mutations driving clonal expansions in spermatogonia (CES) are also transmitted to the next generation. This results in an effective increase of *de novo* mutation rate for CES drivers^5–8^. CES was originally discovered through extreme recurrence of *de novo* mutations causing Apert syndrome^5^. Here, we develop a systematic approach to discover CES drivers as hotspots of human *de novo* mutation. Our analysis of 54,715 trios ascertained for rare conditions^9–13^, 6,065 control trios^12,14–19^, and population variation from 807,162 mostly healthy individuals^20^ identifies genes manifesting rates of *de novo* mutations inconsistent with plausible models of disease ascertainment. We propose 23 genes hypermutable at loss-of-function (LoF) sites as candidate CES drivers. An additional 17 genes feature hypermutable missense mutations at individual positions, suggesting CES acting through gain-of-function (GoF). Among candidates are 5 of 13 known CES drivers^7,8^, 13 drivers of somatic expansions, and 21 members of major signaling pathways; notably, 17 genes show CES evidence in direct sperm sequencing^21^. CES increases the average mutation rate ~17-fold for LoF genes in both control trios and sperm and ~500-fold for pooled GoF sites in sperm. Positive selection in the male germline elevates the prevalence of genetic disorders and increases polymorphism levels, masking the effect of negative selection in human populations. Despite the excess of mutations in disease cohorts for 19 LoF CES driver candidates, only 9 show clear evidence of disease causality^22^, suggesting that CES may lead to false-positive disease associations.

## Introduction

The genome of the average newborn harbors ~70 *de novo* point mutations, of which ~80% arise in paternal germline^14,23^. *De novo* mutation is a common cause of sporadic monogenic disease^9–13,15^, which has led to multiple international efforts collecting large cohorts of parent-child trios^9–13,15^. These trios typically include unaffected parents and their children who, depending on the cohort, may be affected by conditions ranging from neurodevelopmental disorders to congenital heart diseases^9–13,15^. To discover the genes associated with disease, these studies compare the observed counts of *de novo* mutations in a gene with the ones expected from a baseline mutation rate model^9,12^. Due to the initial ascertainment on disease phenotype, this signal provides evidence for the gene’s causal role.

Studies of *de novo* variation, most prominently studies of sporadic neurodevelopmental disorders (NDD)^9^ and autism spectrum disorders (ASD), have identified hundreds of disease genes. As expected, the vast majority were found to be under strong negative selection consistent with a significant impact of *de novo* mutations on severe early-onset conditions^9^. However, we observe that a small subset of the identified genes defy a simple dependence of disease effect and selection and exhibit substantial levels of population polymorphism. Another observed discrepancy is that counts of specific high-impact *de novo* mutations in disease trio cohorts exceed the maximal levels explainable by disease ascertainment. A potential explanation is that forces beyond random mutagenesis increase the observed incidence of mutations.

**Figure 1 |:**
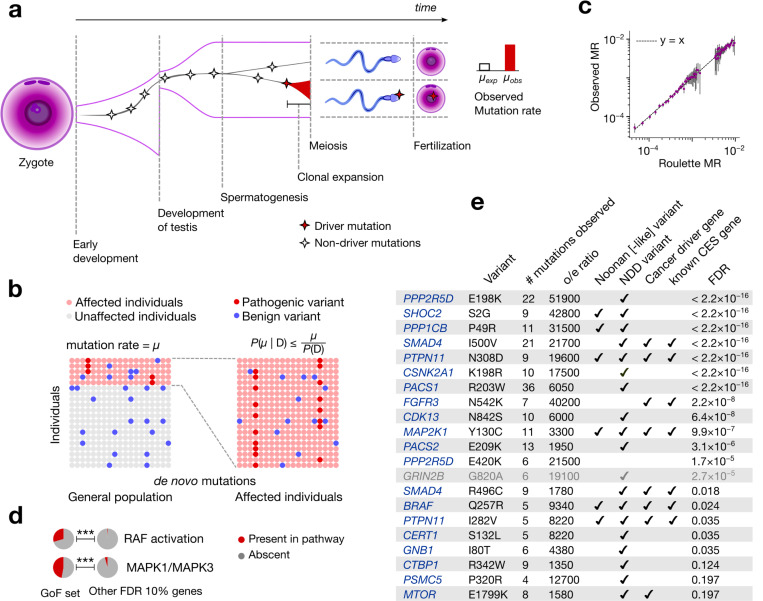
Clonal expansions in spermatogonia and disease ascertainment. **(a)** Effect of clonal expansions in sperm on the observed mutation rate **(b)** In a randomly sampled cohort of individuals affected by a specific disease, the frequency of any variant is elevated due to ascertainment by at most the inverse of disease prevalence. P(D) is disease prevalence and μ is the mutation rate **(c)** Observed synonymous *de novo* variant counts in the neurodevelopmental disorder (NDD) cohort published^9^ by Kaplanis et al., 2020 stratified by Roulette mutation rate bins vs. Roulette predictions. 95% Poisson confidence intervals are shown. **(d)** Fractions of genes included in Reactome RAF activation and MAPK1/MAPK3 pathways. P-values of Fisher’s exact test for comparison of fractions below 0.001 are shown as triple asterisks (***). **(e)** CES driver candidates due to GoF.

Many genes within this subset are involved in carcinogenesis or clonal expansions in somatic tissues suggesting that a plausible explanation may be offered by the positive selection leading to clonal expansions in spermatogonia (CES). Clonal expansions have been recently found to be a feature of all renewing tissues^1–4^, including spermatogonia. Indeed, gain-of-function (GoF) mutations in 13 genes^7,8,^ have already been demonstrated to drive CES. In contrast to somatic tissues, CES increases the likelihood of transmitting driver variants to the next generation, elevating the observed mutation rate in the offspring (Fig. 1a). For this reason, the first CES drivers were discovered through their recurrence in patients with monogenic disease^5,6^. By increasing mutation rate, CES can lead to an increased prevalence of disease. Conversely, genes that drive CES without directly causing disease could appear as disease-causing due to the elevated number of *de novo* mutations observed in affected children.

To systematically investigate the possible effect of CES on human germline mutation and identify potential CES driver candidates, we developed a statistical approach leveraging human *de novo* mutation data^9–15,23^ along with population genetic variation resources^20^. Most of the identified genes follow biological expectation for drivers of clonal expansions such as involvement in MAPK^7^ and other major signalling pathways. We validated our findings using the already discovered CES genes^7,8^, cohorts of unaffected trios^12,14–19^ and comparison with the results of the direct sperm sequencing^21^.

## Results

Rates of DNA damage, together with imperfections of DNA repair and replication determine the baseline *de novo* mutation rate in the male germline^24^. Positive selection in spermatogonia elevates observed mutation rates of the drivers relative to the baseline (Fig. 1a). The approaches to identify CES genes taken here will generally compare observed mutation counts to those expected under a model of baseline mutation rate.

Historically, mutation rates at functional sites have been estimated from the prevalence of phenotypes caused by mutations at those sites. We generalized this logic to identify CES genes in a large trio sequencing cohort ascertained by any phenotype. Specifically, we counted *de novo* mutations in the largest assembled trio cohort ascertained by neurodevelopmental disorder (NDD) phenotype^9^ (31,058 affected probands). The effect of ascertainment has a strict upper bound given by the inverse prevalence of ascertained phenotype:

(1) $$\frac{\text{\#}obs}{\text{\#}exp}\approx \frac{P(V|D)}{P(V)}=\frac{P(D|V)}{P(D)}\leq \frac{1}{P(D)},$$

where P(V) is the probability of *de novo* variants defined by a mutation rate model (Supplementary Text 1) and P(D) is the overall disease prevalence (Fig. 1b). Unlike disease-causing genes with no CES effect, CES drivers could violate this upper bound, offering an approach for finding CES drivers. We develop a statistical approach based on Eq. 1 and demonstrate that it is robust to non-monogenic inheritance (Supplementary Text 4).

From the biological standpoint, because many known CES driver mutations cause NDD^7,8^, we may expect the uncharacterized CES drivers to be similarly prone to causing NDD. The count of CES-driving mutations in the NDD cohort should be greater than in an unascertained set of trios, maximizing the chance of identifying CES drivers involved in NDD. Genes that drive CES but do not cause NDD, can be found by this approach only if the effect of CES significantly exceeds the maximal effect of disease ascertainment given by the inverse prevalence (Supplementary Text 4).

This approach is sensitive to misspecifications of the mutation rate model. For the mutation rate model Roulette^25^, we demonstrated the accuracy of predictions for the synonymous variants in the NDD cohort (Fig 1c, Methods-5 ).

Precision of the prevalence estimates, P(D), might also affect the applicability of Eq. (1). To address this issue we adopted a conservative lower bound on prevalence^26^ of NDD of 1% (see Methods-6.2). We also show that the phenotypic sampling is uniform across the NDD subcohorts ruling out false positive CES findings due to heterogeneous ascertainment by phenotype (Methods-6.1).

### CES explains hypermutability of putative gain-of-function mutations

**Extended Data Figure 1 |:**
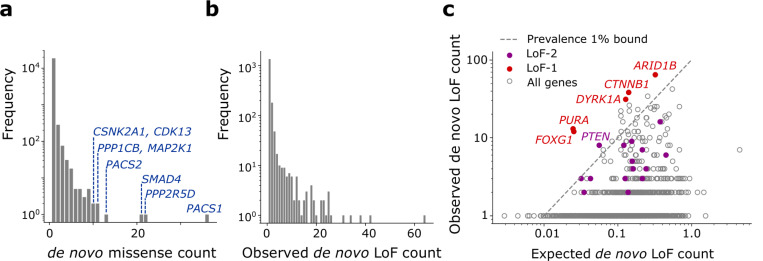
Counts of *de novo* variants in the NDD cohort. **(a)** Numbers of *de novo* missense variants stratified by recurrence. Genes harboring variants occurring >10 times in the cohort are shown in blue. **(b)** Numbers of *de novo* loss-of-function variants aggregated by gene stratified by recurrence. **(c)** Scatter plot of observed vs. expected *de novo* loss-of-function variant counts in the NDD cohort. LoF-1 set genes are shown in red; LoF-2 set genes are shown in purple. Upper bound of disease ascertainment in Eq (1) given by the lower bound of prevalence of 1% is shown as a dashed line.

**Extended Data Figure 2 |:**
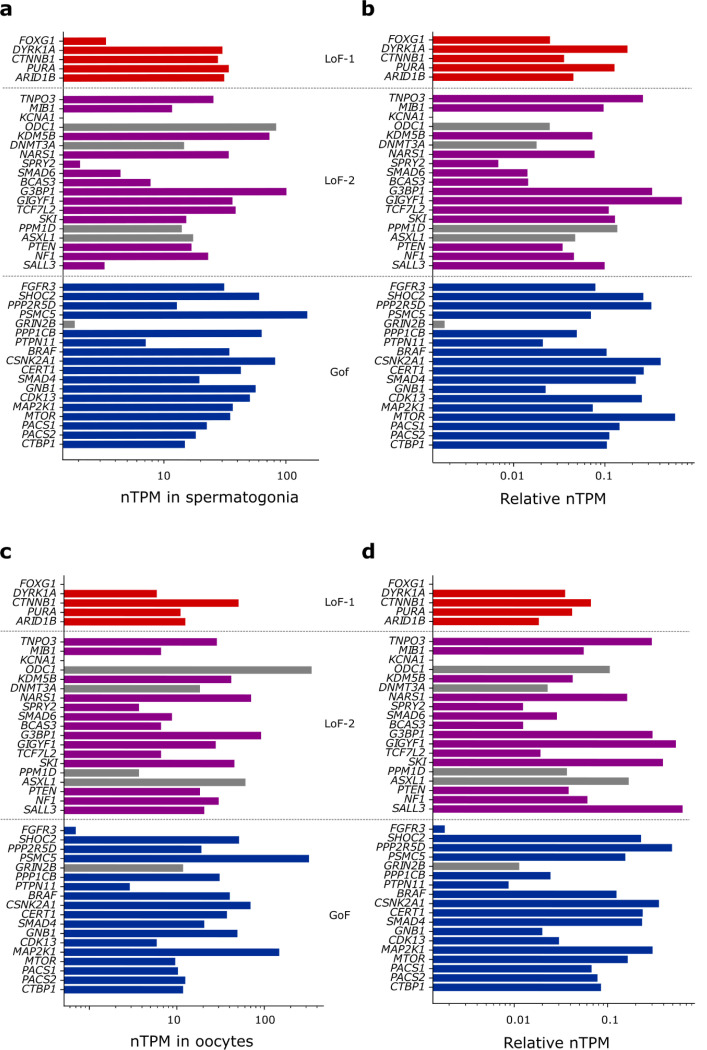
Expression of the identified CES drivers in germline tissues. **(a)** nTPM values for spermatogonia reported in The Human Protein Atlas single-cell dataset. **(b)** nTPM values normalized by the maximal expression across all tissues for each gene. **(c)** nTPM values in oocytes. **(d)** Normalized nTPM values in oocytes.

All CES genes discovered to date act through gain-of-function (GoF) mechanisms, and all known CES mutations are individual missense variants^7,8^. We therefore first apply Eq. (1) to all possible (5×10^7^) individual missense *de novo* mutations in the NDD cohort and identify 21 variants in 18 unique genes that pass the 20% FDR threshold (Extended Data Figure 1a, Suppl. Tab. S11). Among the 18 identified genes, 5 have been previously experimentally established to cause CES^7,8^. From the functional perspective, the identified genes are enriched in RAF activation (adjusted p=1.7×10^−7^) and MAPK1/MAPK3 (Fig 1d, adjusted p=2.2×10^−7^) pathways^27^ in agreement with previous studies^7^ (Suppl. Tab. S12, see Methods-7). All but one of these genes are expressed in spermatogonia (p=6.5×10^−7^). The exception, *GRIN2B,* was thus excluded from the GoF set (Extended Data Figure 2) as a potential false positive.

As explained above, the test based on Eq. 1 has greater power to identify CES drivers that also cause NDD. Indeed, 16 out of 17 genes have independent evidence of association with disease phenotypes through gain-of-function variants (Fig. 1e).

### CES explains hypermutability of loss-of-function mutations

**Figure 2 |:**
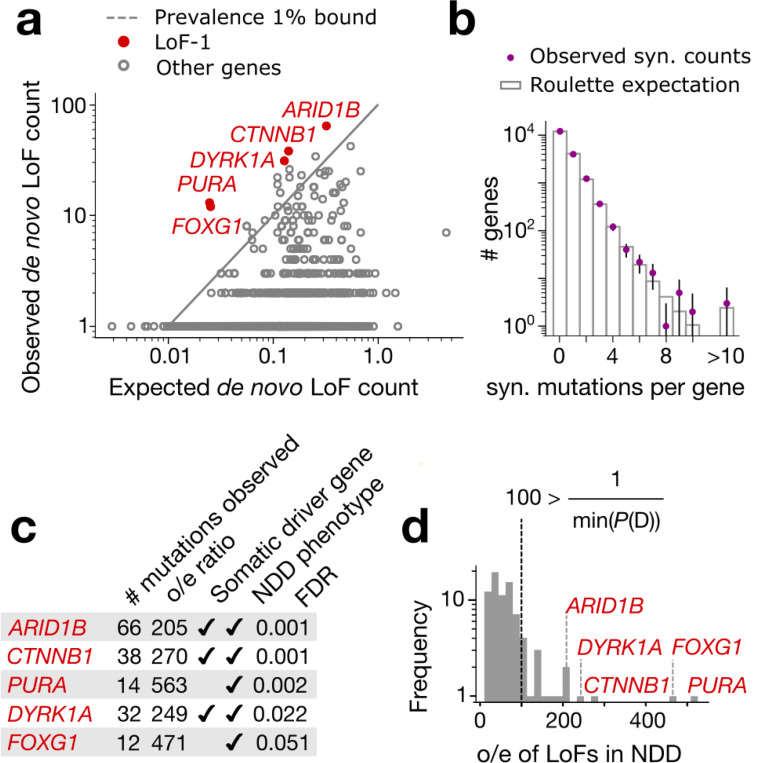
LoF-1 set of putative CES drivers. **(a)** Observed vs. expected LoF counts in the NDD cohort. LoF-1 genes are shown in red. **(b)** Observed synonymous *de novo* mutation counts in the NDD cohort vs. the ones expected under the Poisson counts around Roulette estimates **(c)** Properties of LoF-1 genes **(d)** Ratio of observed to expected *de novo* LoF variant counts (o/e) for genes at FDR < 0.1 in the neurodevelopmental disorder (NDD) cohort published^9^ by Kaplanis et al., 2020. The outliers with the ratio above the maximal ascertainment (LoF-1 genes) are highlighted in red.

Although existing experimental work has focused solely on GoF mutations driving CES, LoF mutations are known to also drive clonal expansions in cancers and healthy renewing tissues^1^. This motivated us to investigate LoF mutations as plausible CES drivers (Fig. 2a). Because every gene harbors multiple possible LoF mutations, we may extend Eq (1) to test sets of variants within genes rather than individual variants. We validated that the aggregation of LoF sites by gene does not bias our mutation rate expectation (Fig. 2b, Methods-5).

Five genes display numbers of de novo LoF mutations in the NDD cohort significantly exceeding any plausible ascertainment by disease (by Eq. 1): *PURA*, *ARID1B*, *CTNNB1*, *DYRK1A* and *FOXG1* (Fig. 2cd, Extended Data Figure 1bc). We call this list the LoF-1 set. Three of them, *ARID1B*, *CTNNB1*, *DYRK1A*, are involved in cancerogenesis or other clonal expansions^28,29^ (Fig 2c). Although *PURA* and *FOXG1* have not been previously identified as drivers of somatic clonal expansions, their functions fit the profile of clonal expansion drivers. *PURA* is involved in replication and transcription control and *FOXG1* is a transcription factor regulating early development (Fig. 2c).

The independent evidence of association with disease phenotypes supports the effect of LoF mutations on NDD for all five of these genes. Also, these genes are highly selectively constrained in the human population (LOEUF < 0.3), consistent with their role in severe pediatric conditions.

### CES and loss-of-function polymorphism

We now bring our attention to CES drivers with weak or no effect on NDD. The approach outlined above is not suited well to identify such genes, because it requires the counts of *de novo* mutations in the NDD cohort to exceed the expectation by at least 100 fold (inverse prevalence of NDD). Therefore, we rely on additional considerations. In particular, we note that CES should increase the rate of functionally consequential mutations and thus polymorphism levels in the general population. The rate of *de novo* mutations in a disease cohort should be elevated for both CES genes and disease genes. However, unlike CES driver with no effect on NDD, disease-causing genes should be deprived of functional polymorphism in the predominantly healthy population. This discrepancy between amounts of genetic variation in the population among genes significant for the excess of *de novo* variants in the NDD cohort over the mutation rate expectation (not necessarily exceeding the boundary of Eq. 1) yields a procedure for identifying CES genes. Specifically, a gene with both large amounts of polymorphism in the general population and the excess of de novo variants in a disease cohort may be deemed a CES driver candidate. Note that this procedure may be applied to cohorts with mixed disease phenotypes, because the expectation is defined just by the mutation rate model and is independent of ascertainment, motivating us to increase power by merging *de novo* variation in cohorts ascertained by NDD (31,058 affected probands)^9^ and autism spectrum disorders (ASD, 16,877 affected probands)^12^. We quantify LoF polymorphism in the general population using LOEUF^20^, an upper bound estimate of the observed-to-expected ratio of LoF SNV counts.

**Extended Data Figure 3 |:**
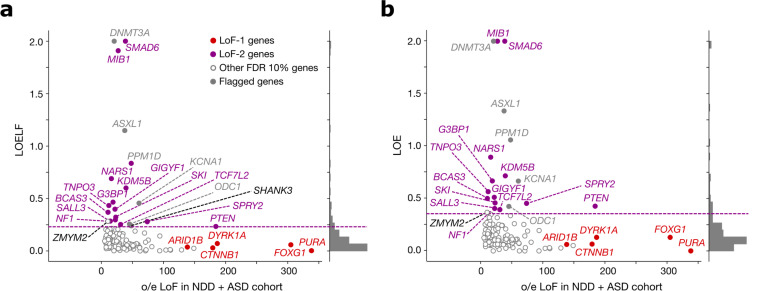
Stability of LoF-2 set with respect to the metric of loss-of-function constraint. **(a)** Observed-to-expected variant count ratio (o/e) for *de novo* LoFs in genes with FDR < 0.1 in the neurodevelopmental disorder cohort (NDD) merged with the autism spectrum disorder (ASD) cohort plotted against the Loss-of-function Observed/Expected Upper-bound Fraction (LOELF) scores. The dashed violet line indicates the minimal LOELF value across LoF-2 genes of 0.23. LoF-2 genes are shown in violet, LoF-1 genes are shown in red, genes above the chosen LOELF threshold but not included in the LoF-2 set (*SHANK3* and *ZMYM2*) are shown in black. **(b)** Same as in (a), but for the Loss-of-function Observed/Expected (LOE) metric. The upper bound for LOE (shown as violet dashed line) is 0.355.

**Extended Data Figure 4 |:**
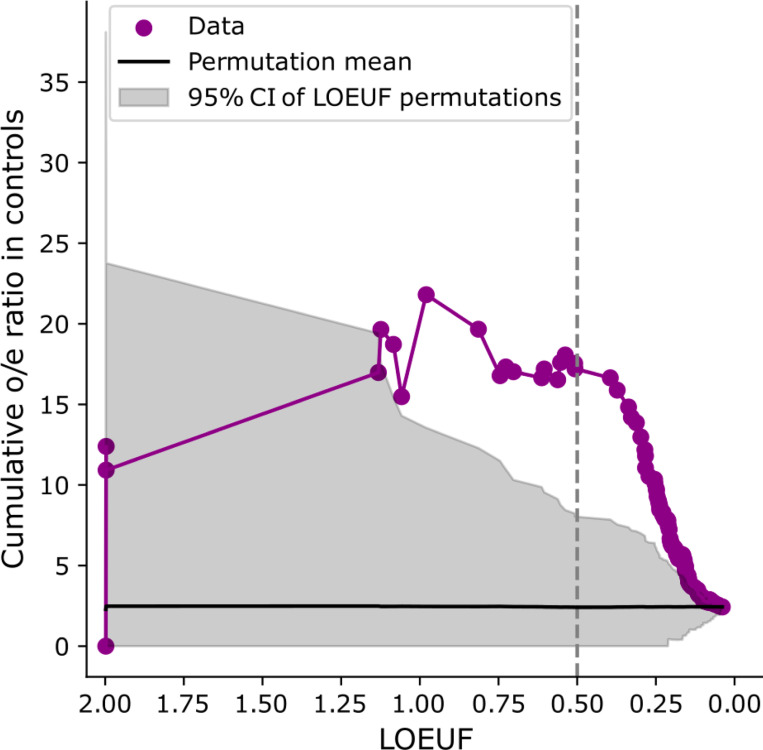
Ratio of observed-to-expected counts of LoF de novo mutations in a cohort of control trios for LoF-2 set genes. The ratio is shown as a function of the LOEUF threshold: we aggregate all genes with LOEUF values lower than the value indicated on the x-axis and calculate the cumulative observed-to-expected ratio. The shaded grey area represents the 95% confidence interval obtained by permuting the LOEUF labels.

**Figure 3 |:**
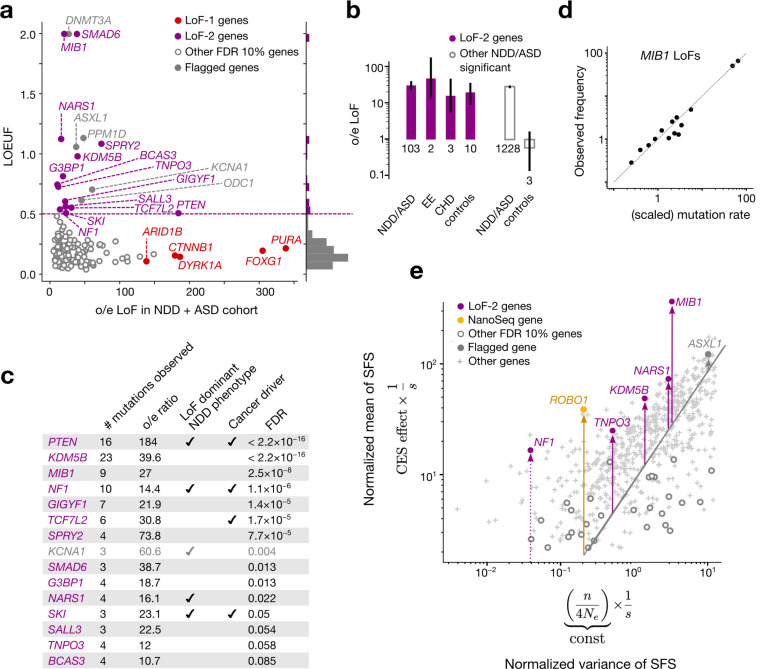
Effect of CES on *de novo* mutations in disease and on the levels of LoF polymorphism in population. **(a)** Observed-to-expected variant count ratio (o/e) for *de novo* LoFs in genes with FDR < 0.1 in the neurodevelopmental disorder cohort (NDD)^9^ merged with the autism spectrum disorder (ASD)^12^ cohort plotted against LOUEF scores. The dashed violet line indicates the 0.5 threshold for LOEUF used to construct the LoF-2 set, and the histogram to the right shows LOEUF values. **(b)** Observed-to-expected LoF variant count ratio in four different trio cohorts. Violet bars indicate LoF-2 genes and empty grey bars indicate other genes significant at FDR < 0.1 in the ASD/NDD cohort. 95% Poisson confidence intervals are shown. Counts of variants are shown below the bars. **(c)** Properties of LoF-2 genes. The single gene not expressed in spermatogonia (*KCNA1*) is shown in grey **(b)** Frequency of LoF in gnomAD-v4 against mean scaled mutation rate predicted by Roulette in *MIB1*. LoF sites were aggregated by mutation rate and each point represents the mean taken for at least 10 sites. **(e)** x-axis: inflation of the variance of the LoF allele frequencies in gnomAD-v4 due to random genetic drift proportional to the inverse selection coefficient (1/s). y-axis: mean allele frequency scaled by mutation rate equal to CES effect multiplied by 1/s. Each point corresponds to an individual gene with at least 10 LoF CpG sites.

122 genes show a significant excess of LoF *de novo* mutations in the ASD/NDD cohort at FDR < 0.1 (see Methods-6.5, Suppl. Tab S7). From these, we selected 19 genes (Fig 3a, Extended Data Figures 3,4) with LOEUF > 0.5 (concordant with cutoffs usually chosen for relaxed negative selection^12,30–32^). High values of LOEUF may be indicative of both relaxed selection and misspecifications of the mutation rate model, as should be the case with CES-driven mutation rate elevation. Genes with misannotated LoF variants^33,34^ or involved in clonal expansions in blood^3^, may also have spuriously high LOEUF values for reasons unrelated to CES. Due to these reasons, four genes were flagged as potential false findings (Supplementary Text 2,3). The remaining 15 genes we call the LoF-2 set.

Four additional considerations support this set of genes as CES driver candidates. First, we show that LoF-2 genes have a significant excess of *de novo* LoF mutations not just in the joint ASD/NDD dataset, but also in a healthy trio cohort^12,14–19^ (Fig 3b). The excess of mutations in the healthy trio cohort could not be affected by disease ascertainment, allowing us to estimate the average CES effect for LoF-2 genes. Surprisingly, the magnitude of the effect in LoF-2 genes is comparable between ASD/NDD and control cohorts (Fig 3b, 18.7 vs 26.9, p=0.2), implying that the effect of disease ascertainment is most likely small and the majority of LoF-2 genes do not cause ASD/NDD. Indeed, only four genes from this set have independent evidence of the causal role in ASD/NDD^22^ (Fig 3c). Just one of these genes, *PTEN,* demonstrates an excess of *de novo* LoF mutations in the NDD cohort comparable to the excesses in the LoF-1 set (Fig 3a, c). In line with small effects of NDD ascertainment in LoF-2 genes, we observe similar magnitudes of the enrichment of *de novo* LoF mutations observed in other trio cohorts ascertained by different phenotypes: encephalopathic epilepsy and congenital heart disease. Finally, using the cohort of healthy controls, we additionally estimated that just 15 LoF-2 genes explain 3.8% of the entire *de novo* LoF variation genome-wide.

To investigate the robustness of the LOEUF threshold used to select genes in the LoF-2 set, we compared the excesses of LoF mutations in the control cohort for different thresholds. The magnitude of the effect does not change substantially in the range of LOEUF threshold values from 0.3 to 2.0 (Extended Data Figure 4). The effect in the healthy cohort dissipates for ASD/NDD genes with LOEUF<0.3, consistent with causal disease effect of these genes.

Second, all LoF-2 genes barring *KCNA1* are expressed in spermatogonia (expression enrichment p = 0.01, Mann-Whitney U test). *KCNA1* was flagged as a potential false-positive finding (Extended Data Figure 2). Four LoF-2 genes are also known to cause cancers^28^.

Third, with the help of population variation data, we can find additional evidence that the high level of LoF polymorphism in LoF-2 genes likely reflects elevated *de novo* mutation rate rather than relaxed selection. The large sample size of gnomAD-v4 (1.6M haploid genomes) enables analysis of frequencies of LoF variants generated by recurrent mutations^25,35–37^. LoF mutations within the same gene are expected to have identical functional effects, including on CES. All individual LoF sites within the same gene therefore share a common linear inflation factor relative to baseline mutation rate expectations. In the presence of CES, allele frequencies remain linearly proportional to the baseline mutation rate attesting to CES being a linear effect, as illustrated by the example of *MIB1* in Fig 3d.

We expand this logic to statistically detect elevation of mutation rate for LoF-2 set in the human population data. Variants with the same mutation rate and selection coefficient have different allele frequencies due to the effect of genetic drift. Strong selection overpowers the effect of drift and makes allele frequencies approach the expectation under the balance between mutation and selection. CES is expected to shift this balance and increase the average allele frequency, but is not expected to affect deviations from this balance. Slightly more formally, the distribution of sampling allele frequencies in the presence of recurrent mutation and relatively strong selection, is given by the Nei approximation^38^ (see Methods-9). Relying on the Nei approximation, we isolate variance inflation due to genetic drift. It is inversely proportional to selection coefficient. The mean allele frequency is similarly inversely proportional to selection coefficient, but is also directly proportional to the effect of CES. Thus, CES effects may be identified from the comparison of the mean and variance terms for a single gene. Even with the current sample sizes, this procedure has low power, as most of the information is contained in frequencies of highly mutable yet rare CpG sites, and so we restricted our analysis to 952 genes with more than 10 CpGs. Fig 3e shows the evidence of CES effects for LoF-2 genes included in the analysis.

**Extended Data Figure 5 |:**
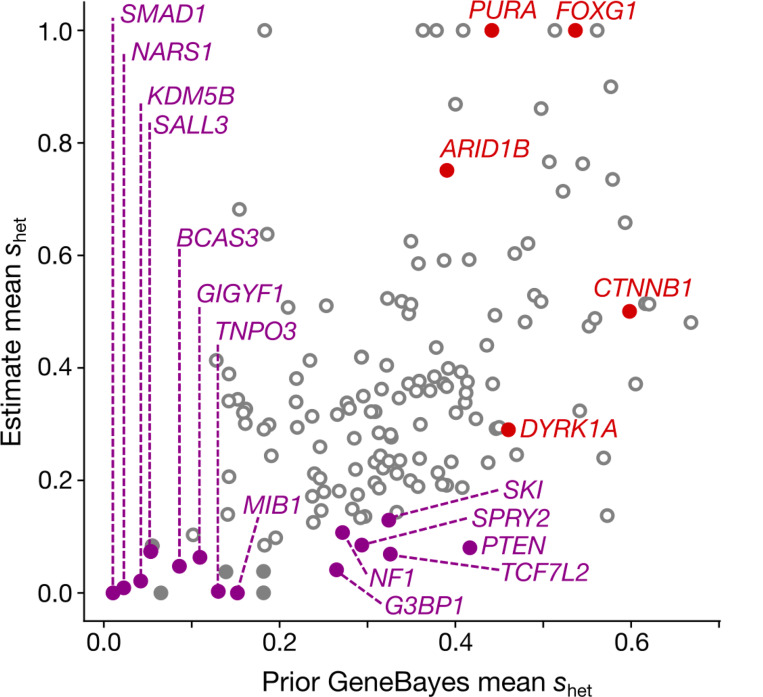
Validation of LoF-2 genes with a non-LOE metric. Prior of the GeneBayes *s*_*het*_ calculated using biological features of genes (x-axis) and the *s*_*het*_ values updated with LoF polymorphism data from gnomAD-v4 (y-axis). See Methods-8 for details.

Our final analysis relies on GeneBayes^39^, which evaluates per-gene selective constraint relying on a protein function-informed prior and a likelihood based on LoF variation within the gene. The prior, unlike the likelihood, is not influenced by mutation rate and the effects of CES. We observe LoF-2 genes have the most inconsistent prior and likelihood estimates, concordant with the effects of CES on the likelihood (Extended Data Figure 5).

### Comparison with direct NanoSeq sperm sequencing

Direct sequencing of mature sperm provides important data for the evaluation of CES effects without any ascertainment by phenotype. In parallel with this study, NanoSeq, an accurate single-molecule resolution DNA sequencing technology, was applied to sequencing sperm cells of 63 donors^21^. A panel of 263 genes implicated in cancer was sequenced with a mean coverage of 8×10^4^ and whole exomes were sequenced with a mean coverage of 2.1×10^4^. Neville et al. identified 40 genes under positive selection in spermatogonia based on enrichment of non-synonymous mutations in sperm^21^. Direct sequencing of sperm is effectively equivalent to assaying paternal *de novo* mutations across all genes in a large cohort of randomly selected trios barring those mutations not compatible with live birth, a case that is discussed below. We used these data to verify some of our predictions.

To evaluate the level of concordance, we tested if CES driver candidates identified in human genetics datasets achieved significance in the test of Neville et al. aggregating all types of non-synonymous mutations^21^. Of all 40 genes identified here, 17 are nominally- (20 at the p-value threshold of 0.1 used by Neville et al.) - and 11 study-wide significant in Neville et al. We compiled the joint list of all putative CES genes between studies (Supplementary Table S20).

**Figure 4 |:**
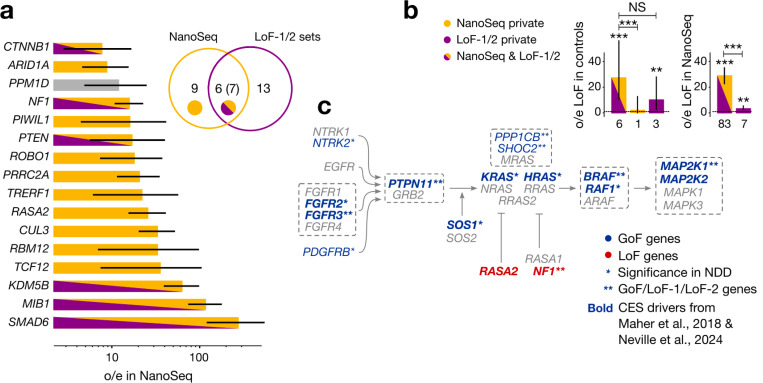
Comparison with direct sequencing data **(a)** Observed-to-expected ratio for LoFs counts in NanoSeq sequencing of sperm for genes with significant excess of LoFs. Poisson-conjugate (Gamma) 95% confidence intervals are shown. Grey bar indicates that *PPM1D* has been flagged in our previous analysis. Venn diagram shows the numbers of genes in sets 1 and 2 of genes significant due to LoFs in our study, number of genes significant due to LoFs in NanoSeq study and the number of common genes. Numbers in parentheses include *PPM1D*. Below, overlaps with set 1 and set 2 genes are shown. **(b)** Enrichments *de novo* mutations in controls for genes overlapping between NanoSeq and LoF-1 + LoF-2, genes private to NanoSeq, and genes private for set 1 + set 2. Number of asterisks indicates the Binomial significance threshold: (*): 0.05, (**): 0.01, (***): 0.001, NS: non-significant. Counts of variants are shown below the bars. **(c)** Core pathway of RAF activation. Genes associated with CES in any of the CES sets are highlighted with blue font and genes with missenses significant in NDD are highlighted with blue shading. Gray dashed frame shows protein complexes.

Next, we compared genes of LoF-1 and LoF-2 sets with 16 genes significant in the NanoSeq dataset exclusively due to LoFs (FDR 20%). As shown in Fig. 4a, out of 16 NanoSeq genes, 7 overlap with our LoF sets.

**Extended Data Figure 6 |:**
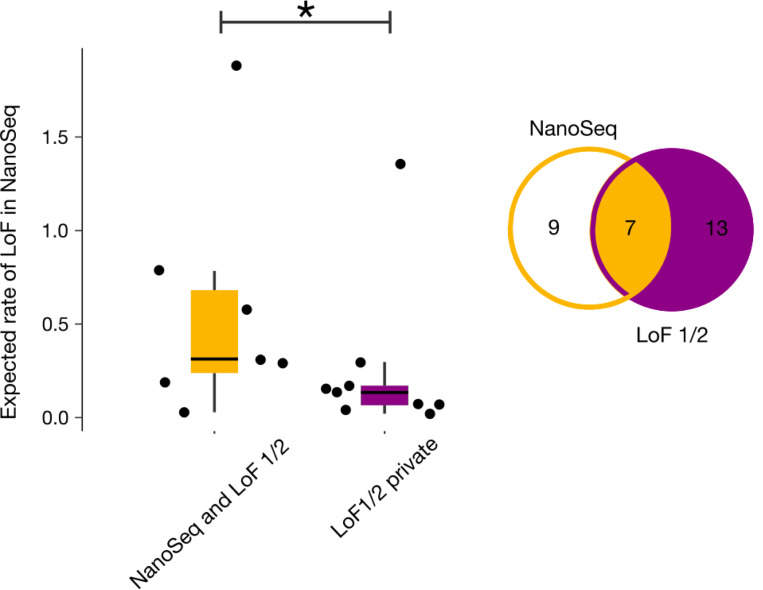
Expected LoF rate in NanoSeq data for LoF-1 and LoF-2 genes. Rates are shown separately for genes overlapping with those significant in NanoSeq and for private LoF-1/2 genes. An asterisk (*) indicates *p* < 0.05 from the Mann–Whitney U test.

Partial overlap between the lists could be attributed to multiple factors. First of all, datasets obtained by NanoSeq, have variable coverage among the genes, and indeed our CES candidates that are not significant in Neville et al, have lower coverage (Extended Data Figure 6). Second, for GoF-1 and LoF-1 the two approaches have differential statistical power. For the human genetics approach, the power is enhanced by the effect of CES drivers on NDD, whereas the power of the NanoSeq experiment is not boosted by the ascertainment. Finally, the power of both approaches is affected by sampling variance in the low mutation count data.

To demonstrate that the overlap is limited by power, we show that a residual CES effect exists in genes outside of the overlap. We calculated the observed-to-expected ratio for LoF mutations in the NanoSeq dataset for genes that did not attain genome-wide significance, but are included in our LoF sets (Fig 4b). We also measured the observed-to-expected ratio for genes outside the overlap using *de novo* variants in control trios (Fig 4b). In both cases, we observe significant enrichment of LoF mutations (p < 0.01, Poisson test).

In the NanoSeq dataset, the combined LoF-1 and LoF-2 gene sets demonstrate a 16.6-fold (Poisson 95% CI 13.5–20.2) excess of LoF mutations. For the GoF set, we observe a remarkable 524-fold excess of mutation counts in sperm sequencing (Poisson 95% CI 311–828).

## Discussion

The unique position of gametogenic tissues as a bridge between somatic and germline evolution allows CES to be viewed from two different perspectives and in terms of two different fields. From the human genetics perspective, CES acts as a factor inflating mutation rate at certain positions. From the biological perspective, CES can be related to cancer with similar genes being involved in clonal expansions in normal tissues and oncogenic transformation.

### Biological and clinical significance of putative CES drivers

Biological functions of many of our CES driver candidates are consistent with the role in clonal expansions (Suppl. Tab S6). We note that the method used here biases the resulting gene sets towards NDD and ASD causal genes, but the functional roles of CES driver candidates are distinct from the bulk of known ASD/NDD genes.

Specifically, out of 40 identified genes, 21 play a role in major signalling pathways (Suppl. Tabs. S6, S15–S18). The pathways include MAPK, WNT and TGF-β (Suppl. Tab. S13). As expected, the MAPK pathway has the highest enrichment (8 genes, Fig. 4c). We noticed that the putative CES drivers are concentrated on the pathway structure and the LoF and GoF annotations are mirrored in the activator/repressor roles (Fig. 4c).

Serine-threonine kinase CSNK2A1 and genes activated by it (PACS1 and PACS2)^40^ form an intriguing group of functionally related CES driver candidates within the GoF set. Another related kinase, CSNK2B^40^, has been reported by Neville *et al.*. R203T mutation in PACS1 is the most recurrent variant in the NDD cohort^41^ and explains 0.1% of all cases and E209K mutation in PACS2 explains another 0.04%. The high recurrence of these variants in the NDD cohort may be explained by their role in both NDD^41,42^ and CES.

As expected for genes involved in clonal expansions, 12 out of 40 genes identified here are COSMIC census cancer drivers^28^. Four of them (*FGFR3*, *PTEN*, *MTOR* and *BRAF*) have been specifically associated with testicular tumors^43–46^.

There is a discrepancy in the average elevations of mutation rate due to CES between GoF and LoF mutations (524-fold vs 17 -fold). We propose two possible explanations. First, spermatogonia cells are diploid, and the variants discussed here are likely heterozygous. LoF mutations are often partially recessive with moderate effects in heterozygotes^47^. In contrast, GoF mutations are usually dominant. The second explanation comes from population genetics. We observe very limited numbers of GoF CES drivers in a single gene (Fig 1e). On the other hand, since all LoF mutations within a gene have identical functional consequences, the number of potential CES driver mutations is large. As CES drivers are frequently involved in severe diseases, the GoF-like 500-fold elevation in mutation rate might generate a substantial rate of *de novo* pathogenic mutations, e.g. is the same inflation was present for LoF mutations in *ARID1B*, about 0.5% of all individuals would be born with NDD if the CES effect was so high. Such pathogenic burden created by LoF mutations in a single gene will trigger efficient negative selection on the CES effect.

### Relationship between CES and developmental diseases

Here, we develop a series of tests to identify CES drivers using the counts of *de novo* mutations in cohorts ascertained by disease, primarily NDD. CES effect and disease ascertainment independently increase the counts of *de novo* mutations in these cohorts. This gives us an upper hand to find CES genes with involvement in NDD as compared to non-ascertained cohorts like trio controls or sperm sequencing. Indeed, out of 40 recovered genes, 26 have orthogonal functional or genetic evidence of involvement in NDD. As expected based on the properties of the tests discussed above, GoF and LoF-1 gene sets are more enriched in genes causal for NDD. This falls in line with the notion of CES determining the prevalence of multiple types of NDD. A well-known example is given by the Noonan syndrome where clonal expansions underlie both etiology and prevalence of the disease. For Noonan syndrome mutations, the effect observed in NanoSeq is a staggering 440-fold (CE 91–1285), which resolves the controversy between the net baseline mutation rate of ~10^−7 and the prevalence of the syndrome of ~10^−4.

**Extended Data Figure 7 |:**
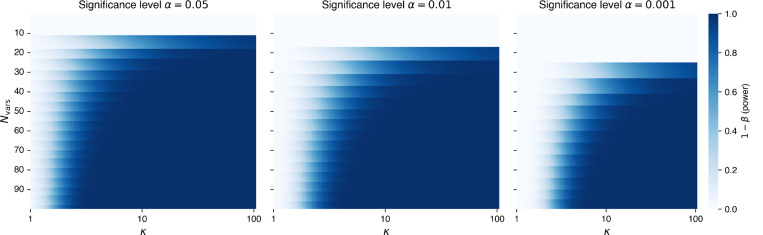
Power analysis of paternal transmission. Statistical power (i.e., the probability of correctly detecting a signal when it exists) of the Binomial test for paternal overtransmission relative to the baseline of 0.75 is shown across the range of CES-related mutation rate inflations *κ* and counts of observed variants. Results are presented for three significance levels: 0.05, 0.01, and 0.001.

On the other hand, many of the recovered CES driver candidates, especially the ones from the LoF-2 set, e.g. *MIB1* or *TCF7L2*, have no strong additional supporting evidence for NDD meaning that they could be false-positives in studies of gene-disease association that rely solely on *de novo* enrichment. However, these studies may find evidence of the causal role in NDD not confounded by CES such as: i. transmission distortion and other types of familial segregation (Extended Data Figure 7), ii. case-control analyses, iii. phenotypic similarity of mutation carriers and iv. functional assays *in vitro* and *in vivo*.

### Other modulators of observed *de novo* mutation rate

Aside from CES and disease ascertainment, one modulator of the observed *de novo* counts is partial lethality (Table 1). It is expected to decrease the observed mutation rates and therefore renders our approaches for finding CES genes conservative. The comparison of *de novo* counts between sperm sequencing and trio sequencing has a potential to highlight lethal mutations. Following this logic, we find that LoF mutations in *RASA2* are consistent with partial embryonic lethality.

Although here we interpret the elevation of mutation rate relative to the baseline as the effect of CES, the mutation rate increase may be due to clonal expansions in early development or in oocyte progenitors. Formally, our approach does not pinpoint the source of the clonal expansions. Still, substantial overlap (given statistical limitations) with sperm sequencing data supports the CES hypothesis. No mutations reported by Neville et al. have appreciable variant allele frequency in sperm^21^, ruling out early developmental origin. Historically, CES is the only reported clonal mechanism responsible for the elevation of germline mutation rate.

In this study we have shown that the phenomenon of CES might be of unexpectedly great importance for future studies in the fields of genetics of rare disease, population genetics, cancer biology and in the emerging field of clonal evolution in somatic tissues.

## Extended Data

## Supplementary Material

Supplement 1

Supplement 2

## Figures and Tables

**Table 1 |:** Different data modalities offer possibilities for distinguishing types of modulators of observed *de novo* mutation rates

	NDD	Clonal expansions			Partially penetrant embryonic lethality
		Spermatogonia	Early development	Oocyte progenitors	
***de novo* effect in cases**	↑	↑	↑	↑	↓
***de novo* effect in controls**	0	↑	↑	↑	↓
**Effect in sperm sequencing**	0	↑	↑	0	0
**Segregation in population**	↓	↑	↑	↑	↓
**Other considerations**					
**Disease phenotype**	yes	no	no	no	no
**Somatic mosaicism**	no	no	yes	no	no
**Parental bias**	baseline	paternal	50/50	maternal	baseline

## Data Availability

All data used in this study, including intermediate processed datasets, are provided in the Supplementary Tables. The full analysis pipeline, including code to reproduce figures and statistical analyses, is available on GitHub at https://github.com/mikemoldovan/CES_Discovery. Associated summary files have also been deposited on Zenodo at https://zenodo.org/records/15660433.
