# Supplementary material for: Cohort-level analysis of human de novo mutations points to drivers of clonal expansion in spermatogonia
Source: medRxiv. 2025 Jun 20:2025.01.03.25319979. Preprint. [Version 2] doi: 10.1101/2025.01.03.25319979 (PMC12204251; doi:10.1101/2025.01.03.25319979)
Supplement: Supplementary file 1 [file media-1.pdf]

Methods

1. Data

We used the dataset of *de novo* variants in probands affected by neurodevelopmental disorders (NDD) published by Kaplanis et al.[9]. For autism spectrum disorders (ASD), we used the *de novo* portion of the SFARI dataset published by Zhou et al.[12]. For congenital heart diseases (CHD), encephalopathic epilepsy (EE) and trios with healthy offspring used as controls, we used aggregated datasets of *de novo* variants in cohorts with probands affected by the respective conditions[10,12-19,48-52]. For any non-NDD and non-ASD cohorts, we filtered out probands with duplicate IDs and samples where *de novo* variants were not obtained by whole genome or whole exome sequencing. Next, for the cohorts with *de novo* variants reported in hg19 coordinates, we mapped the coordinates to hg38 using LiftOver[53].

A recently published NanoSeq dataset of sperm cell sequencing was downloaded from the supplementary materials of Neville et. al., 2024 and candidates due to LoF were taken as genes with the LoF enrichment at the FDR < 0.2 level given in the original table (`qtrunc_cv` column).

COSMIC cancer tiers of the putative CES genes and observed recurrence of variants in these genes with respect to cancer association, were obtained from COSMIC database on October 25th, 2024[28]. The list of genes causing clonal expansions in hematopoetic tissues was obtained from Bernstein et al., 2024[3].

Data on single-cell gene expression in sperm progenitors and oocytes were downloaded from The Human Protein Atlas (REF XXX) and data on single-cell RNAseq of early human embryos was obtained from the Supplementary information of the study by Xu et al., 2023 XXXREF. The structure of the MAPK pathway shown on Fig. 4d was obtained from WikiPathways/WP382[54]. For the per-site mutation rate estimates and quality control tracks, we used Roulette-MR and Roulette-QUAL features of Roulette, respectively[25].

2. Processing of genes

Since Roulette mutation rates are available only for autosomes, we restricted all analyses to autosomal genes. To avoid ambiguity in assigning variants, we further limited our dataset to genes with non-overlapping coding sequences (CDS). For any pair of genes with overlapping CDS regions, we retained the longer gene and excluded the shorter one. In cases where more than two genes overlapped, we selected the largest subset of non-overlapping genes that maximized the total CDS length. This filtering yielded a final set of 17,791 autosomal protein-coding genes, listed in Supplementary Table S14.

3. Processing of variants

We included only coding variants with a "high" or "TFBS" quality score in the Roulette-QUAL track. For each gene, variants were assigned to one of three functional categories. First, loss-of-function (LoF) variants were defined as those with both LOFTEE high-confidence annotations[57] and a VEP consequence of `stop_gained`, `splice_donor_variant`, or `splice_acceptor_variant`. Second, synonymous variants were defined as those with a VEP annotation[55] containing only the term "synonymous," excluding any variants [possibly] involved in splicing. Third, we included missense variants only if they were annotated as "missense" in VEP and had AlphaMissense[56] scores greater than 0.1.

4. Mutational expectation

To scale mutational expectations in an unbiased way, we normalized by the observed number of synonymous *de novo* mutations. This approach accounts for cohort-specific factors such as average parental age and sequencing coverage. For a given variant v, we define the expected number of occurrences in a *de novo* dataset as:

$$\lambda_v := N_S \frac{\mu_v}{\sum_{v \in S} \mu_v},$$

where N_S is the total number of synonymous *de novo* mutations observed in the cohort, S is the set of all admissible synonymous variants, and μ_v is the unscaled Roulette mutation rate assigned to v. This formulation reflects the assumption that *de novo* mutation events occur independently, allowing expected counts to be proportional to the summed rates.

For any annotation $A = \{v\}$, the expected number of *de novo* mutations is:

$$\lambda_A := \sum_{v \in A} \lambda_v = N_S \frac{\sum_{v \in A} \mu_v}{\sum_{v \in S} \mu_v}. \tag{1}$$

This enables comparison of observed and expected mutation counts across functional categories while controlling for mutation rate variation and technical biases.

5. Control for Biases in Roulette

To test for overdispersion in Roulette estimates at the single-variant level, we compared two models of counts of *de novo* synonymous mutations in the NDD cohort: a Poisson model assuming no overdispersion, and a Negative Binomial model assuming overdispersion by a factor of $\gamma + 1$. Using expected mutation counts defined as in Eq. (1), the likelihood under the Poisson model is:

$$\mathcal{L}_0 = \prod_{v \in S} e^{-\lambda_v} \frac{\lambda_v^{n_v}}{n_v!}, \tag{2}$$

where n_v is the observed number of recurrent *de novo* mutations at site v. The likelihood under the Negative Binomial model, with variance inflated by a factor of $\gamma + 1$, is:

$$\mathcal{L}_\gamma = \prod_{v \in S} \binom{n_v + \frac{\lambda_v}{\gamma} - 1}{n_v} \left(\frac{\gamma}{\gamma + 1}\right)^{n_v} \left(\frac{1}{\gamma + 1}\right)^{\frac{\lambda_v}{\gamma}}, \tag{3}$$

using the parametrization of the Negative Binomial distribution by mean λ_v and overdispersion factor γ, such that the variance is $\lambda_v(1+\gamma)$. Since Eq. (3) can be rewritten as a Poisson–Gamma mixture, λ_v/γ need not be an integer.

After maximizing the likelihood in Eq. (3) over γ, we obtain $\hat{\gamma} = 0.004$. Comparing the log-likelihoods of Eq. (2) and Eq. (3), we find a difference of $\log \mathcal{L}_\gamma - \log \mathcal{L}_0 = 31.1$. Applying the Akaike Information Criterion (AIC), which penalizes additional parameters by 2 log-likelihood units, this strongly supports the presence of modest but statistically significant overdispersion.

As a result, we use the Negative Binomial distribution with $\gamma = 0.004$ rather than the Poisson model when modeling individual mutations in the GoF gene set.

Beyond single-site effects, Roulette estimates might have regionally correlated errors resulting in some regions having generally over- or underpredicted mutation rates. In particular, correlated errors in residuals could systematically distort the expected counts for specific regions of interest, such as protein-coding genes and may be undetectable at the per-site level. To assess this, we tested for overdispersion in the number of synonymous *de novo* mutations aggregated by gene. Let n_g be the observed and λ_g the expected count of synonymous mutations in gene g, with λ_g obtained by summing site-specific rates as in Eq. (1). We then substitute $n_v \to n_g$ and $\lambda_v \to \lambda_g$ in Eq. (2) and Eq. (3), and re-estimate the log-likelihoods.

In this gene-level analysis, the log-likelihood difference is $\log \mathcal{L}_\gamma - \log \mathcal{L}_0 = 0.69$, which falls well below the AIC threshold for including an extra parameter. Thus, we find no evidence of significant variance inflation at the gene level.

To further test for overdispersion in the gene-level expectations λ_g, we used a regression-based approach. Applying the law of total variance:

$$\mathrm{Var}(n_g) = \mathbb{E}[\mathrm{Var}(n_g \mid \lambda_g)] + \mathrm{Var}[\mathbb{E}(n_g \mid \lambda_g)],$$

where the first term captures residual (Poisson) noise and the second term reflects underlying variation in λ_g. Since $\mathbb{E}(n_g \mid \lambda_g) = \lambda_g$ and $\mathrm{Var}(n_g \mid \lambda_g) = \lambda_g$ under the Poisson model, we expect:

$$\mathrm{Var}(n_g) = \mathbb{E}(\lambda_g) + \mathrm{Var}(\lambda_g).$$

To test this, we computed sample estimates of each term and tested the Poisson assumption with the chi-squared goodness-of-fit test on statistic

$$\chi^2 = \sum_g \frac{(n_g - \lambda_g)^2}{\lambda_g},$$

with degrees of freedom equal to the number of genes minus 1 (17,790). The resulting p-value of 0.6 confirms consistency with the Poisson model, with no evidence of excess dispersion. We thus conclude that gene-level Roulette residuals are effectively uncorrelated, and that variance inflation in gene-level mutational expectations is negligible.

Consequently, for gene-level aggregation of variant counts in LoF-1 and LoF-2 sets, we used Poisson tests to assess enrichments.

6. Statistics

6.1. Phenotypic homogeneity in the NDD cohort

Our procedures yielding LoF-1 and GoF sets of putative CES drivers assume uniform phenotypic sampling across the NDD cohort (see Main Text and below). Deviations from uniformity—e.g., overrepresentation of certain syndromes—could result in differential ascertainment of associated mutations, potentially inflating effect sizes for certain genes and introducing false positives to LoF-1 and LoF-2 sets.

To assess homogeneity of recruitment in the composite NDD cohort used here, we compared contributions of the three sub-cohorts (DDD, GeneDx and RUMC) comprising it to *de novo* LoF

variation in individual genes. If the cohorts were sampled in a uniform way, the fractions of *de novo* variants coming from each cohort across all genes should correspond to the per-cohort mutation rate, which is proportional to the number of synonymous mutations per individual cohort (Eq. 1). The numbers of variants in each gene should therefore come from the Multinomial distribution with probabilities given by per-cohort synonymous variant counts and the number of events given by the number of observed *de novo* variants.

We tested this hypothesis by comparing four models:
1. A Multinomial model with probabilities fixed by observed synonymous variant counts (0 free parameters).
2. A Multinomial model with unconstrained probabilities to test for shifts in representation (2 free parameters due to 3 probabilities which need to sum up to 1).
3. A Dirichlet-Multinomial model centered on the observed synonymous proportions, allowing for overdispersion (1 free parameter: amplitude of Dirichlet).
4. A fully flexible Dirichlet-Multinomial model accounting for both overdispersion and shifted means (3 free parameters: 2 for unconstrained Multinomial and 1 additional parameter for the amplitude of Dirichlet).

For each model, we computed the total likelihood across all genes and compared models using the Akaike Information Criterion (AIC). The unbiased Multinomial model (Model 1) was the one preferred by AIC, indicating that the observed gene-level LoF variant distributions are consistent with uniform cohort sampling.

To further validate that our LoF-1 and LoF-2 sets are not confounded by non-uniform sampling, we repeated the analysis on specifically LoF-1 and LoF-2 putative CES drivers. In both cases, the AIC again favored the null Multinomial model. Together, these results suggest that recruitment across the DDD, GeneDx, and RUMC cohorts was phenotypically homogeneous with respect to LoF variant ascertainment. Full likelihood and AIC values for all model comparisons are reported in Supplementary Table S2.

6.2. Prevalence of NDD

The identification of putative CES drivers in the LoF-1 and GoF sets relies on the assumption that the disease prevalence estimate $P(D)$ used in Eq. 1 (Main Text) is not inflated. Overestimating $P(D)$ would lead to underestimating the maximum plausible effect size attributable to ascertainment, thereby increasing the risk of false positive CES calls in highly penetrant genes.

Epidemiological estimates of neurodevelopmental disorder (NDD) prevalence—based on diagnoses of intellectual disability or global developmental delay—typically fall within the range of 2–3%[26]. To ensure a conservative interpretation, we imposed a lower bound of 1% on $P(D)$, which corresponds to an upper bound of 100 on the effect of ascertainment.

One additional consideration suggests that 1% is an appropriate lower boundary for NDD prevalence. Almost all significantly mutated genes have the effects below 100 with very few genes greatly exceeding this threshold (Fig. 3d). The possibility that the prevalence is even lower than 1% and the observed distribution of gene effects is fully explained by disease ascertainment is inconsistent with the existence of genes with substantial penetrance for NDD.

6.3. Construction of the GoF set

To define the GoF set of putative CES driver genes, we applied a Negative Binomial test. The expected count of *de novo* variants at position v was set to $\mu_v/P^*(D)$, where μ_v is the Roulette mutation rate at v, and $P^*(D)$ is the lower bound on disease prevalence, fixed at 0.01 (see

Main Text). To account for overdispersion at the single-site level, we used a variance inflation parameter $\gamma = 0.004$, as defined in Eq. (3). For multiple testing correction, we applied a Bonferroni adjustment using a correction factor equal to the total number of autosomal missense variants annotated by VEP and filtered to have an AlphaMissense score above 0.1 (49,686,008 tests).

6.4. Construction of the LoF-1 set

To define the LoF-1 set of putative CES driver genes, we applied a Poisson test to LoF *de novo* variants observed in the NDD cohort. The expected count of variants in gene g was set to $\lambda_g/P^*(D)$, where λ_g is the LoF mutation rate for gene g as defined in Eq. (1), and $P^*(D)$ is the lower bound on disease prevalence, fixed at 0.01 (see Main Text). As in the GoF analysis, this expectation corresponds to the number of events expected under maximal disease ascertainment (i.e., full penetrance). The choice of the Poisson model is justified in the "Control for Biases in Roulette" section above. For multiple testing correction, we used a Bonferroni factor equal to the number of autosomal genes with at least one filtered LoF site (17,791 tests). This yielded five Bonferroni-significant genes at the 0.05 level, and one additional gene (*FOXG1*) significant at FDR < 0.1, using the Benjamini–Hochberg procedure (Fig. 2ad).

6.5. Construction of the LoF-2 set

The LoF-2 set of putative CES driver genes was constructed using the combined ASD + NDD cohort in two steps. First, we identified genes with a significant excess of *de novo* LoF variants relative to expectation ($n_g > \lambda_g$), where n_g is the observed LoF count and λ_g is the Roulette-based expectation for gene g. As in the LoF-1 analysis, we used a Poisson test with correction for for 17,791 tested autosomal genes (Supplementary Table S14). On the second step, we defined the LoF-2 set as those genes passing a false discovery rate threshold of FDR < 0.1 and having gnomAD v4 LOEUF scores greater than 0.5 (Fig. 2a).

6.6. Excess of LoF Variants in NDD Compared to NanoSeq

To test whether the excess of *de novo* LoF variants observed in the NDD cohort exceeded that in the NanoSeq sperm dataset, we used a Binomial test. For each gene g, we set the probability of success to the expected fraction of LoF mutations arising from NanoSeq, defined as $\frac{\lambda_{m,g}}{\lambda_{m,g}+\lambda_n}$, where $\lambda_{m,g}$ is the expected LoF count in NanoSeq and λ_n is the expected LoF count in NDD. The number of trials was set to $n_g + m_g$, where n_g and m_g are the observed LoF counts in gene g in the NDD and NanoSeq datasets, respectively. The test compared the observed fraction $\frac{m_g}{m_g+n_g}$ to the expected null fraction. Only genes with at least one LoF variant in both datasets were tested. A gene was deemed significant if its P-value, Bonferroni-corrected for the number of tests ($N = 5$), was below 0.05. This test identified only *CTNNB1* and *PTEN* as significantly enriched for LoF variants in the NDD cohort relative to NanoSeq. Since the NanoSeq donors were older on average than the parents of individuals in the NDD cohort, this comparison is conservative. The test for the excess of variants in NanoSeq compared to the excess in NDD cohort highlights *CUL3* and *ARID1A* as genes with a larger excess in NanoSeq. Although this effect may be confounded by the age differences between the parents in the NDD cohort and the donors of samples used in the NanoSeq experiment, an alternative explanation may be lethality of LoF variants in these genes.

7. Expression and Gene Set Enrichment Analyses

Expression analysis of candidate genes was performed using single-cell data from The Human Protein Atlas. For each gene, the Atlas reports TPM expression values across a wide range of tissues. To account for gene-specific differences in baseline expression, we normalized the TPM values in spermatogonia and oocytes by the maximum TPM observed for each gene across all tissues. To statistically assess differences in expression patterns between CES driver candidates and other genes, we applied the Mann–Whitney U test to the normalized TPM values.

Gene set enrichment analysis was performed using the g:Profiler[27] web service with default parameters. We tested five gene sets: the GoF, LoF-1, and LoF-2 sets individually; the union of LoF findings (LoF-1 and LoF-2); and the union of all three sets. Full g:Profiler results are provided in Supplementary Tables S15-S18.

8. GeneBayes s_{het} update and CES

To obtain estimates of LoF constraint in the LoF-2 set of genes unimpacted by mutation rate misspecification we used prior estimates of s_{het}, the selection coefficient against heterozygotes, produced by the GeneBayes method. These estimates result from a prior distribution fit to all LoF variation using a set of global gene features and should be minimally impacted by variation in any one gene[39]. We re-estimated s_{het} for all genes using gnomAD-v4. To assess the robustness of the resulting list of genes to the constraint estimation, we additionally performed an orthogonal analysis of differences between prior and posterior GeneBayes shet estimates, which were obtained on gnomAD-v3. As expected under moderate selection against LoF mutations and CES, prior estimates of s_{het} were substantially higher than those estimated using polymorphism alone. The rationale here is that, given downwardly biased mutation rate estimates of CES genes, the data should shift the prior down by factors larger than those expected for other genes. This analysis recapitulates our results obtained with LOEUF.

Due to the demographic complexity of the gnomAD v4 sample, we used a data-driven approach to estimate s_{het} for each gene. We first fit how both the proportion of polymorphic sites as well as the shape of the site frequency spectrum depend on s_{het}, conditional on μ. For this, we fit a multinomial model to the observed allele counts using prior estimates of s_{het} as a covariate. To ensure that the SFS was not too sparse at higher allele counts, we binned sites by allele count with boundaries $[0, 1, 2, 3, 4, 5, 6, 7, 8, 9, 10, 11, 16, 24, 36, 100]$. The observed distribution of alleles counts in each Roulette mutation rate bin was then transformed to multinomial coefficients such that

$$\log \frac{P(K = i \mid \mu)}{P(K = 0 \mid \mu)} = \beta_i^{\mu} \tag{4}$$

This model for the neutral SFS in each bin was first estimated using synonymous SNV counts. Next, we fit a model to all LoF polymorphism such that

$$\log \frac{P(K = i \mid \mu, s_{het})}{P(K = 0 \mid \mu, s_{het})} = \beta_i^{\mu} - \beta_i^{s} \sqrt{s_{het}}. \tag{5}$$

The β_i^{μ} coefficients were determined by the synonymous SFS and β_i^{s} coefficients were fit with maximum likelihood using the `L-BFGS-B` algorithm as implemented in scipy. The square root dependency on s_{het} was determined by manual inspection of the SFS, binned by s_{het} and μ. The s_{het} values used to fit this global multinomial model were prior mean values output by GeneBayes. This procedure yielded a model for the SFS in each mutation rate bin, given a value of s_{het}. We

then re-estimated s_{het} for LoF mutations in each gene using this model using maximum likelihood and bounding s_{het} to $[0, 1]$.

9. Variants in LoF-2 Genes Segregate in the Population Due to Elevated Mutation Rate

LoF-2 genes, despite showing a significant excess of LoF mutations in the NDD cohort, harbor many LoF variants segregating in the general population. This is reflected, for instance, in their relatively high LOEUF values. The number of segregating sites in a gene is governed by both mutation rate and selection. If these genes are genuine CES drivers, we expect an elevated mutation rate to contribute to the number and frequency of LoF SNVs. In such cases, both the number of segregating sites and their allele frequencies would appear higher than expected under a fixed selection coefficient.

In a cohort with over a million chromosomes, this hypothesis can be evaluated by comparing the empirical expectation and variance of LoF allele frequency distributions across genes. While both mutation rate and selection influence the mean and variance of allele frequencies, recurrent mutation produces alleles that tend to be more common but exhibit lower variance in frequency, due to reduced genetic drift. In contrast, relaxed selection increases both mean and variance via enhanced drift. Thus, we expect genes with elevated mutation rate to show lower variance in allele frequency relative to their mean than genes under relaxed selection.

To formalize this, we applied the Gamma-Poisson approximation for allele frequency spectra under strong selection and recurrent mutation[38].

Gene Filtering. For this analysis, we retained only genes with at least 10 LoF sites passing Roulette-QUAL QC and having unscaled Roulette mutation rate $\mu > 1$ (e.g., CpG>TpG transitions). This filtering ensures reliable estimates of the mean and variance of allele frequencies for each gene.

Model. We assume a three-step generative model for LoF allele counts in each gene:

1. Mutations occur randomly at any LoF site in the gene.
2. Each derived variant segregates in a diploid population of effective size N_e, experiencing negative selection of intensity s in heterozygotes. We assume strong selection and no lineage interference.
3. A sample of n alleles is drawn from the population.

Let k_v denote the number of observed derived LoF variants v in the population sample. In what follows, we will drop the index v. We assume the variance of k to be the sum of contributions of the three assumed steps without any additional terms:

$$\text{Var}(k) = \text{Var}_{\text{sampling}}(k) + \text{Var}_{\text{seg}}(k) + \text{Var}_{\mu}(k), \tag{6}$$

where the components correspond to mutation heterogeneity across sites, drift-induced segregation, and Poisson sampling noise, respectively. Under the Gamma approximation introduced by Nei (1968), the distribution of allele frequencies at LoF sites is modeled with $4N_e\mu$ as the shape parameter and $1/(4N_e s)$ as the scale parameter. The expectation of k is then:

$$\mathbb{E}(k) = \frac{n\,\mathbb{E}(\mu)}{s}.$$

And the first two terms of (6) are given by the variance of the Poisson and Gamma components, respectively:

$$\mathrm{Var}_{\mathrm{sampling}}(k) \equiv \mathbb{E}(k) = \frac{n\,\mathbb{E}(\mu)}{s},$$
$$\mathrm{Var}_{\mathrm{seg}}(k) = \frac{n\,\mathbb{E}(\mu)}{s}\frac{n}{4N_e s}.$$

The final term of (6) may be obtained by noticing that $\mathrm{Var}_{\mathrm{sampling}}(k)+\mathrm{Var}_{\mathrm{seg}}(k) = \mathbb{E}_\mu[\mathrm{Var}(k \mid \mu)]$. Applying the law of total variance,

$$\begin{aligned}
\mathrm{Var}(k) &= [\mathrm{Var}_{\mathrm{sampling}}(k) + \mathrm{Var}_{\mathrm{seg}}(k)] + \mathrm{Var}_\mu(k) \\
&= \mathbb{E}_\mu[\mathrm{Var}(k \mid \mu)] + \mathrm{Var}_\mu[\mathbb{E}(k \mid \mu)] \\
&= \frac{n\,\mathbb{E}(\mu)}{s}\left(1 + \frac{n}{4N_e s}\right) + \left(\frac{n}{s}\right)^2 \mathrm{Var}(\mu) \\
&= \frac{n\,\mathbb{E}(\mu)}{s} + \frac{n\,\mathbb{E}(\mu)}{s}\frac{n}{4N_e s} + \left(\frac{n}{s}\right)^2 \mathrm{Var}(\mu), \\
\mathrm{Var}_\mu(k) &= \left(\frac{n}{s}\right)^2 \mathrm{Var}(\mu).
\end{aligned}$$

We now model the effect of CES as a multiplicative factor $\kappa \geq 1$ on the mutation rate so that:

$$\mathbb{E}(k) = \kappa \times \frac{n\,\mathbb{E}(\mu)}{s}, \tag{7}$$
$$\mathrm{Var}(k) = \kappa \times \frac{n\,\mathbb{E}(\mu)}{s}\left(1 + \frac{n}{4N_e s}\right) + \kappa^2 \times \left(\frac{n}{s}\right)^2 \mathrm{Var}(\mu). \tag{8}$$

The final term in Equation (8) containing dependency on κ^2 can be estimated from the regression of k on μ with Poisson error. Subtracting this term from $\mathrm{Var}(k)$ leaves only the sample estimate of $\mathbb{E}_\mu[\mathrm{Var}(k \mid \mu)]$.

Estimating Selection. This framework yields two estimators of $1/s$:

1. From the mean allele count (Eq. 7):

$$\frac{\kappa}{\hat{s}} = \frac{\bar{k}}{n\bar{\mu}}, \tag{9}$$

   where $\bar{\mu}$ and $\bar{k}$ are the mean mutation rate and mean LoF allele count per gene.

2. From the conditional variance (Eq. 8), subtracting sampling noise:

$$\frac{\mathrm{Var}_{\mathrm{seg}}(k)}{\mathrm{Var}_{\mathrm{sampling}}(k)} = \frac{\mathbb{E}_\mu[\mathrm{Var}(k \mid \mu)] - \mathrm{Var}_{\mathrm{sampling}}(k)}{\mathrm{Var}_{\mathrm{sampling}}(k)} = \frac{n}{4N_e s}. \tag{10}$$

Note that only the first estimator (Eq. 9) depends on κ and is therefore sensitive to CES effects.

Figure 3e plots the log estimate of $\frac{n}{4N_e s}$ (excess variance due to drift) against the log estimate of $\frac{\kappa}{s}$ (elevated mean allele count due to CES). Because $\log(n/4N_e) - \log(s)$ is a constant offset

from $\log(\kappa) - \log(s)$, genes with elevated mutation due to CES appear as positive residuals from the regression line.

This approach is conservative: the Gamma-Poisson approximation is invalid for genes under weak or no selection, where allele frequency variance is even higher. Thus, such genes would not mimic the CES signal.

True NDD genes deviate negatively from the regression line, having lower mean allele frequencies than predicted under a fixed s. This is consistent with either sampling bias in gnomAD v4 resulting from difficulties in recruitment of individuals affected with NDD or from selection acting against LoF variants in these genes even prenatally, leading to inherently reduced representation in population datasets.

MIB1 Example. For *MIB1*, we used Poisson regression to quantify the dependence of LoF allele frequency on mutation rate. To visualize this relationship (Figure 3d), we binned sites by scaled mutation rate, merging bins with fewer than 10 sites with the next-highest bin.

References

48. EuroEPINOMICS-RES Consortium, Epilepsy Phenome/Genome Project & Epi4K Consortium. De novo mutations in synaptic transmission genes including DNM1 cause epileptic encephalopathies. *Am. J. Hum. Genet.* **95**, 360–370 (2014).
49. Helbig, K. L. *et al.* Diagnostic exome sequencing provides a molecular diagnosis for a significant proportion of patients with epilepsy. *Genet. Med.* **18**, 898–905 (2016).
50. Heyne, H. O. *et al.* De novo variants in neurodevelopmental disorders with epilepsy. *Nat. Genet.* **50**, 1048–1053 (2018).
51. Klöckner, C. *et al.* De novo variants in SNAP25 cause an early-onset developmental and epileptic encephalopathy. *Genet. Med.* **23**, 653–660 (2021).
52. Allen, A. S. *et al.* De novo mutations in epileptic encephalopathies. *Nature* **501**, 217–221 (2013).
53. Perez, G. *et al.* The UCSC Genome Browser database: 2025 update. *Nucleic Acids Res.* **gkae974** (2024). doi:10.1093/nar/gkae974
54. Agrawal, A. *et al.* WikiPathways 2024: next generation pathway database. *Nucleic Acids Res.* **52**, D679–D689 (2024).
55. McLaren, W. *et al.* The Ensembl Variant Effect Predictor. *Genome Biol.* **17**, 122 (2016).
56. Cheng, J. *et al.* Accurate proteome-wide missense variant effect prediction with AlphaMissense. *Science* **381**, eadg7492 (2023).
57. `https://github.com/populationgenomics/loftee`
